# Pulmonary nodular lymphoid hyperplasia in a 53-year-old man with malignant sign: a case report

**DOI:** 10.1186/s13019-021-01672-y

**Published:** 2021-10-09

**Authors:** Zhen Yang, Lianshuang Wei, Xu Li, Xin Liu

**Affiliations:** 1 Department of Thoracic Surgery, The Second Affiliated Hospital of Kunming Medical University, 374th Dianmian Road, Kunming, Yunnan Province China; 2 Department of Pathology, De Hong Zhou Ren Ming Yi Yuan, 13th Yonghan Street, Manshi, 678400 China

**Keywords:** Pulmonary nodular lymphoid hyperplasia, Malignant signs, Misdiagnosis, Volume doubling time, Case report

## Abstract

**Background:**

Pulmonary nodular lymphoid hyperplasia (PNLH) is a rare benign illness. Due to atypical clinical and radiographic presentations, diagnosis largely depends on postoperative pathological examination. Thus, preoperative misdiagnosis is often occurred.

**Case presentation:**

We present a case of asymptomatic PNLH that was seen as ground-glass opacity (GGO) on computed tomography (CT). After 3-year observation, the diagnosis tends to adenocarcinoma owing to increasing density of the node and vessel convergence sign, which were signs of malignancy. Video-assisted segmentectomy (S10) was carried out. Histopathologic examination of postoperative specimen showed follicular lymphoid hyperplasia with interfollicular lymphoplasmacytosis, consistent with PNLH. The follow-up chest CT images showed no recurrence or metastasis.

**Conclusion:**

Although it is a benign disease, PNLH can exhibit malignant signs in the imaging examinations, which could lead to misdiagnosis. This reminds us of the uncertainty between imaging findings and diagnosis. The diagnosis depends on postoperative pathological examination. Volume doubling time is a potential parameter to differentiate PNLH from lung cancer.

## Background

Pulmonary nodular lymphoid hyperplasia (PNLH) is a rare benign disorder of the lungs, which was initially proposed known as “pseudolymphoma” in 1983, only a few dozens of cases have been reported since then. The clinical and radiographic presentations can be atypical, thus, misdiagnosis may occur before surgery. In the study, we report a case misdiagnosed as adenocarcinoma because of malignant signs in the CT scan, however, it identified as PNLH by the pathologic findings after operation. It reminds us of the uncertainty between imaging findings and diagnosis. Surgical resection is not only diagnostic but also curative.

## Case presentation

**Fig. 1:**
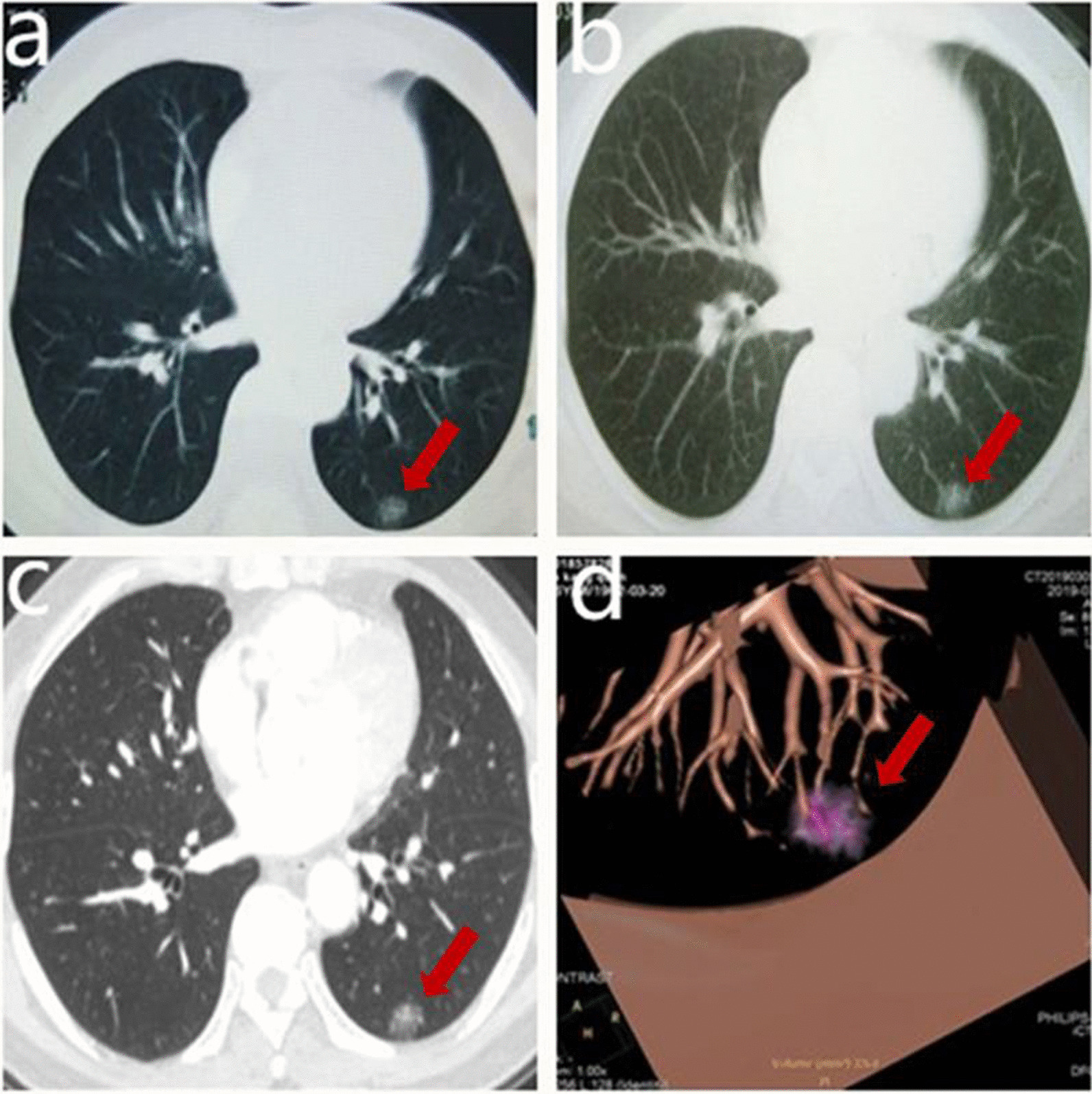
The CT scan of the patient. **a** Initial CT scan performed in July 2017. **b** Follow-up CT scan performed in November 2018. **c** Follow-up CT scan performed March 2019. **d** The three-dimensional reconstruction of CT scan performed in March 2019

A 53-year-old man was transferred from a local hospital after undergoing a routine health examination. He was healthy with no obvious discomfort, and he had 40 pack years smoking history. The initial chest CT scan performed in July 2017 revealed a GGO measuring up to 1.6 cm in maximum diameter and 2.14 cm^3^ in volume in the lower lobe of the left lung (Fig. 1a). The GGO was multifocal, suggesting that it was likely a chronic lesion. A follow-up chest CT scan was performed 16 months later, and no interval change was found (Fig. 1b).

**Fig. 2:**
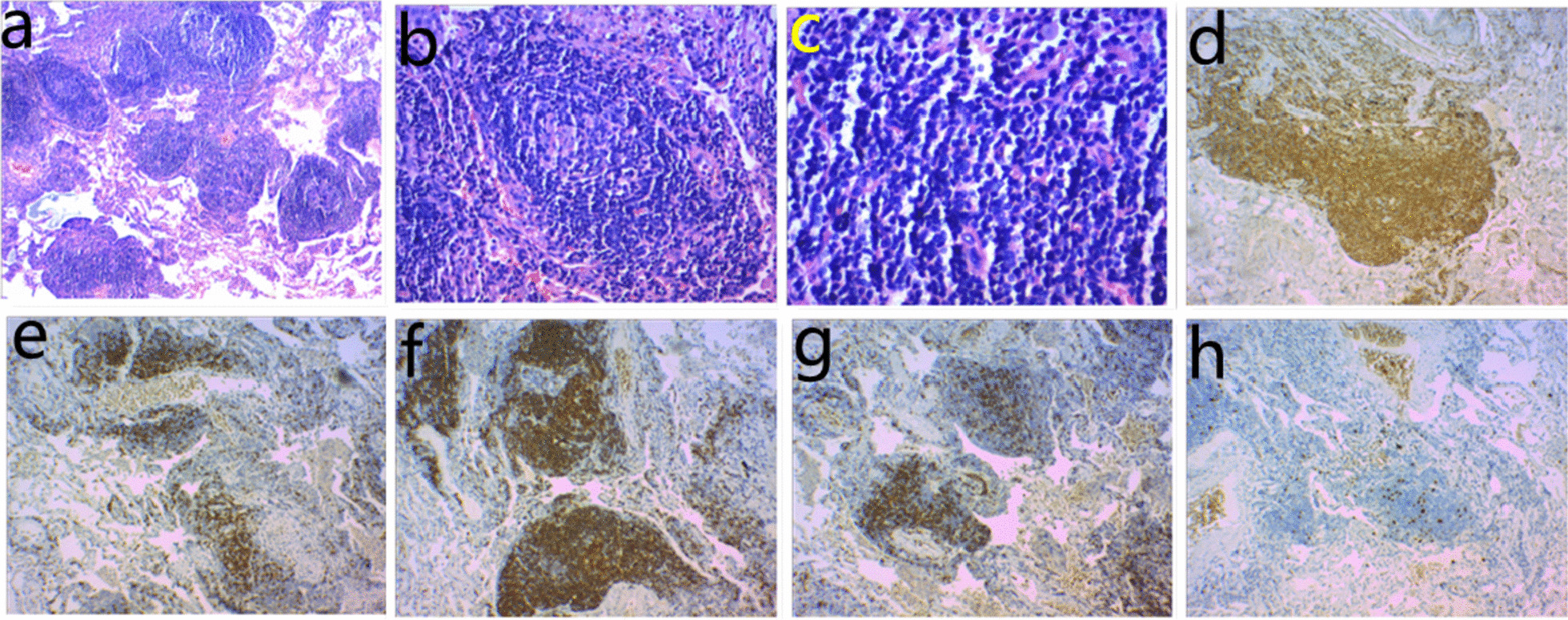
Histopathologic and immunohistochemical result. **a** Reactive germinal centers. **b** Germinal center cells. **c** A mixture of polyclonal B, T lymphocytes and plasma cells. **d** Bcl-6 immunostaining. **e** CD3 immunostaining. **f** CD60 immunostaining. **g** CD45R0 immunostaining. **h** Ki67 immunostaining

Another CT scan performed 20 months later in March 2019 showed density of the GGO increasing (Fig. 1c) and vessel convergence emerging (Fig. 1d), which were signs of malignancy. While the maximum diameter was 1.7 cm and volume was 2.57 cm^3^ in CT scan. Adenocarcinoma seemed to be the most likely diagnosis, followed by AAH. While the maximum diameter and volume of the lesion remained relatively stable. Video-assisted segmentectomy (S10) and lymph node samplings were carried out in March 2019. The resected lung tissue measured 6 × 2.5 × 1 cm, and an ill-defined grayish solid nodule measured 1.2 × 1.1 × 0.8 cm was included. No malignant cells were found on intraoperative frozen section examination. Histopathologic examination showed follicular lymphoid hyperplasia with interfollicular lymphoplasmacytosis, consistent with PNLH. The immunohistochemical results showed positivity for CD3, CD20, CD38, CD45RO, CD68, CD138, PAX-5, and Bcl-6, and negativity for CD23 and Bcl-2 (Fig. 2). No mediastinal lymph node metastasis was found in the specimens.

The patient recovered and discharged 4 days after surgery without any complications. He was diagnosed as PNLH, and subsequently followed-up with chest CT scans regularly. The scans showed no recurrence or metastasis, and the patient did not have any further discomfort, which indicated 48-month follow-up outcome was excellent. Long-term follow-up will be continued.

## Discussion

Pulmonary nodular lymphoid hyperplasia is a reactive lymphadenia, composed of reactive nodular lymphoid proliferation that formed one or more pulmonary masses, usually asymptomatic [1]. No gender-based differences were found in PNLH patients, and most of them were middle-aged and elderly, with the age ranging from 25 to 72 years [2]. PNLH is a rare benign disorder of the lungs, since its discovery, only a few dozen cases have been reported. Thus far, the largest study on PNLH with 67 patients was reported by Likui et al. [2].The specific etiology of PNLH is still not established, although it could be the local bronchial mucosa-related lymphoid tissue’s response to potential antigenic stimulation. It may also be associated with combined variable immunodeficiency or Sjögren syndrome and granulomatous-lymphocytic interstitial lung disease such as IgG4-related sclerosing diseases [3]. In addition, Bégueret et al. highlighted that smoking may also be an important risk factor for PNLH [4].

Because of the rarity, radiological findings can only be gathered from case reports and radiographic descriptions. CT scans show a unique 2–4-cm nodule in most cases, which could be solitary, or multi-nodular, solid, or subsolid, and occasionally with lobulation, vessel convergence, pleural indentation, and mediastinal or hilar lymph node involvement [3], moreover, some authors presented a case of PNLH with positive fluorodeoxyglucose (FDG) uptaking [5]. With these signs of malignancy, lung cancer is the first suspected diagnosis. Thus, misdiagnosis can also occur before pathological examination. Histologically, PNLH is composed of well-defined lymphoid tissue masses with numerous reactive germinal centers, interfollicular lymphocytes, and plasma cells, and is mainly located in the subpleural area. Immunohistochemically, CD3-positive T cells and CD138-positive plasma cells are reactive in the interfollicular zone; whereas, CD20-positive B expresse within CD21-positive follicular dendritic cell meshworks, and do not express Bcl-2 [3]. Our report is in accord with the study above, so PNLH is the diagnosis, in addition, CD20, CD38, CD45RO, CD68, PAX-5, and Bcl-6 were positive, while CD23 was negative, which deserve further study.

Surgical resection is not only diagnostic but also curative. Thus far, although a case suggested the possibility of spontaneous regression of the remaining lesions after resection of the PNLH lesion [6], and a case reported antibiotic-induced reduction of abnormal lung shadow in PNLH [7], few evidence thus far has suggested that PNLH could regress without operation. The surgical approach include lobectomy and sublobar resection; the latter was found suitable to treat PNLH owing to its advantages of enhanced recovery after surgery (ERAS) and preferable long-term outcome [2]. The trail No. JCOG0802/WJOG4607L suggested that segmentectomy is a potentially standard surgical approach for the lung tumor with maximum diameter ≤ 2 cm and consolidation tumor ratio > 0.5 [8], so we chose segmentectomy, and the 48-month follow-up outcome was excellent. The scans showed no recurrence or metastasis, and the patient had no complaints. Long-term follow-up will be continued.

In our case, the lesion emerged as mixed ground-glass opacity, and presented malignant characteristics including increasing density and the vessel convergence sign after follow-up visit, which misled us to diagnose the condition as cancerous before operation. However, during the 20-month watching and waiting period, the maximum diameter and volume of the lesion remained relatively stable, the volume doubling time was 2427 days by calculating with the formula VDT = (t*log2)/(log[Vt/V0]) [9]. Tumor doubling time less than 400 days represents malignant potential [10], in our case the volume doubling time is far more than 400 days, so we deduce the disease is a indolent lesion and volume doubling time is a potential parameter to differentiate PNLH from lung cancer. The assumption is necessary to be validated in a large and reliable dataset.

## Conclusion

Although it is a benign disease, PNLH can exhibit malignant signs in the imaging examinations, which could lead to misdiagnosis. This reminds us of the uncertainty between imaging findings and diagnosis. The diagnosis depends on postoperative pathological examination. Volume doubling time is a potential parameter to differentiate PNLH from lung cancer.

## Data Availability

The datasets used and/or analyzed in the current article are available from the corresponding author on reasonable request.

## Abbreviations

PNLH: Pulmonary nodular lymphoid hyperplasia
GGO: Ground-glass opacity
CT: Computed tomography

## References

[1] Sim J, Koh HH, Choi S, Chu J, Kim TS, Kim H, Han J (2018). Pulmonary nodular lymphoid hyperplasia with mass-formation: clinicopathologic characteristics of nine cases and review of the literature. J Pathol Transl Med.

[2] Fang L, Xu J, Wang L, He Z, Lv W, Hu J (2019). Pulmonary nodular lymphoid hyperplasia: a rare benign disease with malignant mask. Ann Transl Med.

[3] Yell M, Rosado FG (2019). Pulmonary nodular lymphoid hyperplasia. Arch Pathol Lab Med.

[4] Bégueret H, Vergier B, Parrens M, Parrens M, Lehours P, Laurent P, Vernejoux J, Dubus P, Velly J, Mégraud F, Taytard A, Merlio J, Mascarel A (2002). Primary lung small B-celllymphoma versus lymphoid hyperplasia: evaluation of diagnostic criteria in 26cases. Am J Surg Pathol.

[5] Yilmaz U, Unsal I, Halilcolar H, Anar C, Yildirim Y, Sanli A, Kargi A (2009). Nodular lymphoid hyperplasia of the lung: the role of positron emission tomography in diagnosis. Tuberk Toraks.

[6] Miyoshi S, Hamada H, Katayama H, Hamaguchi N, Irifune K, Ito R, Ohtsuki Y, Yoshino T, Higaki J (2010). A case of pulmonary nodular lymphoid hyperplasia with a resected cavity, followed by spontaneous regression of the remaining lesions. Intern Med.

[7] Tanino A, Tsubata Y, Hamaguchi S, Sutani A, Nagase M, Isobe T (2020). Antibiotic-induced reduction of abnormal lung shadow in pulmonary nodular lymphoid hyperplasia. Respirol Case Rep.

[8] Eguchi T, Sato T, Shimizu K (2021). Technical advances in segmentectomy for lung cancer: a minimally invasive strategy for deep, small, and impalpable tumors. Cancers.

[9] Setojima Y, Shimada Y, Tanaka T, Shigefuku S, Makino Y, Maehara S, Hagiwara M, Masuno R, Yamada T, Kakihana M, Ohira T, Ikeda N (2020). Prognostic impact of solid-part tumour volume doubling time in patients with radiological part-solid or solid lung cancer. Eur J Cardiothorac Surg.

[10] Obayashi K, Shimizu K, Nakazawa S, Nagashima T, Yajima T, Kosaka T, Atsumi J, Kawatani N, Yazawa T, Kaira K, Mogi A, Kuwano H (2018). Prognostic impact of a ground glass opacity component in clinical stage IA non-small cell lung cancer. J Thorac Dis.

